# Low Prevalence of *Chlamydia trachomatis* Infection in Non-Urban Pregnant Women in Vellore, S. India

**DOI:** 10.1371/journal.pone.0034794

**Published:** 2012-05-02

**Authors:** Navjyot K. Vidwan, Annie Regi, Mark Steinhoff, Jill S. Huppert, Mary Allen Staat, Caitlin Dodd, Rida Nongrum, Shalini Anandan, Valsan Verghese

**Affiliations:** 1 Cincinnati Children’s Hospital Medical Center, Cincinnati, Ohio, United States of America; 2 Christian Medical College, Vellore, Tamil Nadu, India; London School of Hygiene and Tropical Medicine, United Kingdom

## Abstract

**Objective:**

To determine the prevalence and risk factors for *Chlamydia trachomatis* (CT) infection in pregnant women and the rate of transmission of CT to infants.

**Methods:**

Pregnant women (≥28 weeks gestation) in Vellore, South India were approached for enrollment from April 2009 to January 2010. After informed consent was obtained, women completed a socio-demographic, prenatal, and sexual history questionnaire. Endocervical samples collected at delivery were examined for CT by a rapid enzyme test and nucleic acid amplification test (NAAT). Neonatal nasopharyngeal and conjunctival swabs were collected for NAAT testing.

**Results:**

Overall, 1198 women were enrolled and 799 (67%) endocervical samples were collected at birth. Analyses were completed on 784 participants with available rapid and NAAT results. The mean age of women was 25.8 years (range 18–39 yrs) and 22% (95% CI: 19.7–24.4%) were primigravida. All women enrolled were married; one reported >one sexual partner; and six reported prior STI. We found 71 positive rapid CT tests and 1/784 (0.1%; 95% CI: 0–0.38%) true positive CT infection using NAAT.

**Conclusions:**

To our knowledge, this is the largest study on CT prevalence amongst healthy pregnant mothers in southern India, and it documents a very low prevalence with NAAT. Many false positive results were noted using the rapid test. These data suggest that universal CT screening is not indicated in this population.

## Introduction

*Chlamydia trachomatis* (CT) is one of the most common genital pathogens worldwide [1]. Strategies to screen, test, diagnose and treat this curable, yet prevalent and indolent disease have been adopted in many countries. Disease in women can include severe outcomes such as pelvic inflammatory disease and infertility. In addition, neonates of infected women may have increased morbidity and mortality. Newborn prematurity and low birth weight is shown to be associated with maternal chlamydia infection. Notably 50–75% of infants born to infected mothers become infected at one or more sites including the conjunctiva, nasopharynx, vagina, and rectum leading to purulent conjunctivitis, pneumonia, and trachoma [2]. Hammerschlag recognized that the most frequent site of perinatally acquired chlamydia infection in US neonates is the nasopharynx with rates as high as 70% and that CT pneumonia can occur in 30% of infants with nasopharyngeal infection [3]. In addition, Rosenman found that of US infants exposed to CT at birth, 8 to 44% had conjunctivitis, and 0 to 17% pneumonia [4].

Most epidemiologic data on CT is from industrialized nations; however, it is important to characterize CT disease from resource limited regions where most infants are born. Scarce information is available on laboratory-confirmed incidence and prevalence of chlamydia infection in otherwise healthy males and females in India. Furthermore, the available Indian data show a wide variation in CT prevalence and methods of laboratory confirmation [5]–[14]. Some studies have found infection rates of Indian women ranging from 3.3% to 33% depending on the population sampled [5]–[14] (Table 1). Most of these studies focus on high risk groups (female sex workers, STI/infertility patients, and HIV-positive women) and were limited by small sample sizes [6], [7], [9]–[11], [13]. In Tamil Nadu, Joyee *et al.* found the prevalence of active genital CT infection in a healthy adult female population by NAAT of the urine was 1.1% (95% CI: 0.4%–1.8%) [8]. However, among symptomatic men and women attending a STI clinic, the prevalence of confirmed CT infection by culture and/or nested polymerase chain reaction (PCR) detecting major outer membrane protein (MOMP) was 30.8% (95% CI: 23.4–38.6%) [9].

**Table 1:** CT Prevalence Studies in India.

Area	Year	Age group	Study population	N	Sample	Test	Prevalence	Ref
Vellore, India	1993	Not available	Pregnant women	273	Endocervical swab	Chlamydiazyme kit (Abbott, USA)	3.3% (95% CI: 1.2%–5.4%)	5
New Delhi, India	1995	Mean Age: 34.9 yrs	Generally healthy women, Gynecological clinic(outpatient department)	257	Endocervical swab	Chlamydiazyme kit (Abbott, USA)	23.3%	14
Mumbai, Inner City	1998	≤35 yrs	Suspected PID, Infertility	446	Endocervical swab	ELISA	0.5%	7
Mumbai	2001	18–42 yrs	Gynecological clinic, complications associatedwith reproductive health	123	Endocervical swab	Chlamydiazyme kit (Abbott, USA)	1.7 to 20% among different risk categories	11
New Delhi	2003	18–40 yrs	Symptomatic women Gynecological clinic	280	Endocervical swab	NAAT	28% (18–25 yrs)	13
New Delhi	2003	19–36 yrs	Pregnant women	350	Endocervical swab	DFA & PCR	18.8% (95% CI 14.76–22.96%)	12
Tamil Nadu, South India	2004	15–45 yrs	Healthy adult population, clinic	1066 (serum)841 (urine)Female samples	Serum, urine	IgM-ELISA,urine NAAT	3.3% ELISA, 1.1% PCR (95% CI: 0.4–1.8%)	8
Chennai	2005	N/A	STI clinic(high risk)	143 men & women	Serum, genital swab endocervical/urethral	Culture/nested PCR (MOMP): CT Serum: IgG	30.8% by nested PCR(MOMP)	9
Aligarh, North India	2009	18–40 yrs	Obstetric clinic, secondary infertility, pregnant women (control subjects)	70	Endocervical swab	Cell culture, ELISA	55%–2°infertility; 5.5% pregnant women	10
Karnataka State,South India	2010	Mean age: 30.7 yrs	Symptomatic women Gynecological clinic	412	Endocervical swab	NAAT	2.6%, vaginal discharge; 2.7% vaginal discharge with clinical cervicitis	6

This table shows a review on Indian data which show a wide variation in CT prevalence and methods of laboratory confirmation.

The prevalence of CT in pregnant women in India has been shown to vary by geographic region. In a study conducted in Vellore, TN India in 1993, a prevalence of CT in pregnant women was found to be 3.3% (95% CI: 1.2%–5.4%) using an enzyme immunoassay (EIA) which detects the presence of chlamydia antigen (Chlamydiazyme® kit, Abbott®,USA) [5]. The authors also reported a higher prevalence of CT in rural women (5.9%) compared to urban women (1.8%) [5]. A 1999 study from New Delhi found the prevalence of CT infection in mid-pregnancy and at labor using the Chlamydiazyme® test to be 17% and 18.6%, respectively [15]. In addition, the study also found neonates born to infected mothers experienced purulent conjunctivitis more frequently than those born to non-infected mothers, 12.5% versus 2.8% (p = 0.04) respectively [15]. Rastogi *et al.* also found a CT prevalence of 18.8% (95% CI: 14.76–22.96%) among 350 pregnant women in New Delhi diagnosed using PCR and DFA [12]. One concern about comparing these studies is that the EIA test is estimated to be only 65–70% sensitive and 90–99% specific compared to nucleic acid amplification test (NAAT) methods. Thus the EIA estimates would be expected to underestimate the true prevalence of infection.

Current obstetric practice in southern India does not include universal prenatal screening for CT. This practice is based on evidence from older local data [5]. In contrast, the CDC recommends that pregnant women in the US be screened for CT during their first prenatal visit; and women who are at increased risk for infection (new or multiple sex partners and those under 25 years of age) should have a repeat screen during the third trimester [16]–[17]. This is based on an estimated CT infection rate of 5% among US women of reproductive age [18]. However, there are no data on the current CT prevalence among healthy pregnant women in Vellore, using more sensitive methods such as NAAT testing.

Furthermore, although there have been only a few studies documenting prevalence of *Chlamydia trachomatis* in southern India, there are no studies documenting the frequency of maternal to child transmission of CT in the Vellore region. The Alexander *et al* study [5] is more than a decade old and only documents the prevalence of maternal CT infection. In addition, since 1993, newer techniques for more accurate detection of CT such as NAAT have evolved and the prevalence of CT may have changed. The Clearview Chlamydia Test Kit® (Inverness, Houston, TX 77038) is one of the few FDA approved rapid diagnostic tests (RDT) for CT but a wide range of sensitivity (53–73%) and specificity (68–99%) on endocervical specimens have been reported when compared to culture or DNA probe assay/NAAT [19]–[23]. Newer NAAT technology using the Roche Amplicor® CT/NG test (Roche Molecular Systems, Inc., Branchburg, NJ 08876) shows higher sensitivity of 93.3% and specificity of 99.7% on endocervical specimens compared to cell culture and RDT [24].

In order to provide data for development of evidence-based guidelines for resource-limited regions, the objectives of our study were to determine the prevalence of CT genital infection in delivering women using highly sensitive NAAT testing and to study the maternal to child transmission of CT in Vellore, India.

### Specific Aims of Study

We aimed 1) to estimate the prevalence of genital CT infection among women presenting for delivery at Christian Medical College Hospital (CMCH) Vellore, India using a NAAT technique 2) to identify risk factors associated with chlamydia infections in pregnant and delivering women in order to guide development of screening policies if needed; and 3) to estimate the prevalence of CT infection at birth and to identify factors associated with maternal-infant transmission.

## Methods

### Ethics Statement

The institutional review boards at both Cincinnati Children’s Hospital Medical Center (CCHMC) and the Christian Medical College Hospital approved the study protocol. Written informed consent was obtained during enrollment for the subject and her neonate. All clinical investigation was conducted according to the principles expressed in the Declaration of Helsinki.

### Study Design

This was a prospective hospital-based observational study.

### Surveillance Area

CMCH, located in Vellore, India served as the surveillance site for this study. Vellore is a city in the Indian state of Tamil Nadu located in tropical southern India midway between the cities of Chennai (formerly Madras) and Bengaluru (formerly Bangalore). Vellore town has a population of 177,230 (2001 Indian census) of which 50.4% are female. CMCH is a private, non-profit, 2000+ bed tertiary referral hospital with primary care clinics (including pediatrics) and serves a diverse population of lower to middle class patients. The CMCH obstetrics department reports over 8,000 deliveries per year.

### Study Population

From April 2009 to January 2010, delivering women were approached and recruited daily by a research staff member during an antenatal outpatient visit, a scheduled labor/induction, or in the postpartum ward. During the antenatal outpatient visit, a physician would send his/her patient, based on the study’s inclusion criteria, to the designated research office for the research assistant to discuss the study with the delivering woman. On the inpatient setting, the research assistant approached subjects daily on the obstetric wards that met inclusion criteria. After the research assistant obtained the subject’s consent to the study, a questionnaire on socio-demographic factors, prenatal history, and sexual history was administered by the research assistant on initial encounter during a private interview using a pen and paper format.

### Enrollment Criteria

The inclusion criteria required that study participants be 18 to 45 years of age; greater or equal to 28 weeks gestation; and planning to deliver at CMCH and use the CMCH Vaccine or Child Health clinic for infant care. Pregnant mothers who had a history of chronic disease including diabetes, congestive heart failure, and renal failure were excluded. Stillborn infants and infants with congenital disorders or malformations were excluded.

### Specimen Collection and Laboratory Testing

Endocervical swabs were collected by physicians or a research assistant during a pelvic exam performed either during an antenatal outpatient visit; prior to a scheduled labor/induction; or in the postpartum ward. The first swab was used to perform the rapid diagnostic test (RDT) and the second for NAAT testing. A third swab was stored frozen at −70°C for future testing. Swabs were collected before use of lubricant or insertion of a prostaglandin tablet for induction to prevent the possibility of inhibition of the test.

Within 48 hours after delivery, neonatal participant data was recorded by the research assistant and a conjunctival swab from the eye and a nasopharyngeal (NP) swab were collected. Swabs were collected after 24 hours in order to decrease the chance that a positive test would represent contamination by maternal secretions. After collection, the swabs were stored in a freezer at −70°C for NAAT testing.

### Lab Methods

#### Rapid Test

The RDT used in the study was the Clearview Chlamydia Test Kit® (Inverness, Houston, TX 77038). This test is a one step immunochromatographic assay for direct antigen detection with an internal positive control indicator. The test was performed in the onsite research lab by study staff according to the package insert instructions.

#### NAAT

Endocervical specimens were stored in a freezer at CMCH at −70°C. The NAAT samples were tested at Y.R. Gaitonade Centre for AIDS Research and Education Laboratory in Chennai, India using the Roche Amplicor® CT/NG test for *Chlamydia trachomatis* (Roche Molecular Systems, Inc., Branchburg, NJ 08876). The test was performed according to the package insert instructions. Internal positive controls all tested positive. In addition, blinded commercial positive controls (n = 5) (Accurun® 341, SeraCare Life Sciences, Inc., Milford, MA 01757) were included among the participant samples sent to the lab. All of these blinded positive controls were reported as positive results.

In addition, 102 samples from vials with remaining endocervical swab specimen had DNA extracted using the QIAamp DNA Minikit® (Qiagen, Hilden, Germany). These extracted samples were shipped, on dry ice, and retested independently in an established US laboratory using the Roche Amplicor PCR® (Roche Diagnostic Systems, Inc., Branchburg, N.J).

### Treatment

Subjects’ positive rapid test results were reported to the attending physicians in both the obstetric and neonatology departments following birth. Treatment decisions were made by the primary physicians.

### Statistical Methods

#### Sample size calculation

For sample size estimations, we used estimated prevalence of CT from previous studies (Table 1). It was assumed, from these studies, that the prevalence in our study population is about 3.3%. Using standard sample size calculations, a sample of 799 is sufficient to detect prevalence up to 9% with precision of 2%. For the neonate samples, a sample of 195 provides 80% power to detect a prevalence of 3% with precision of 2.5%.

#### Statistical analysis

Data were analyzed using Statistical Analysis Systems software (SAS Institute, Cary, NC, Version 9.2). Means and standard deviations were used to characterize normally distributed data. T-tests were used to compare means of normally distributed data. Chi-square and Fisher’s exact tests were used to assess differences in proportions. All reported P values were 2-sided and p values <0.05 were considered statistically significant. Concordance between the rapid test and NAAT was determined by the Kappa statistic and Kendall’s Tau B.

## Results

### Prevalence in Pregnant Women

**Figure 1:**
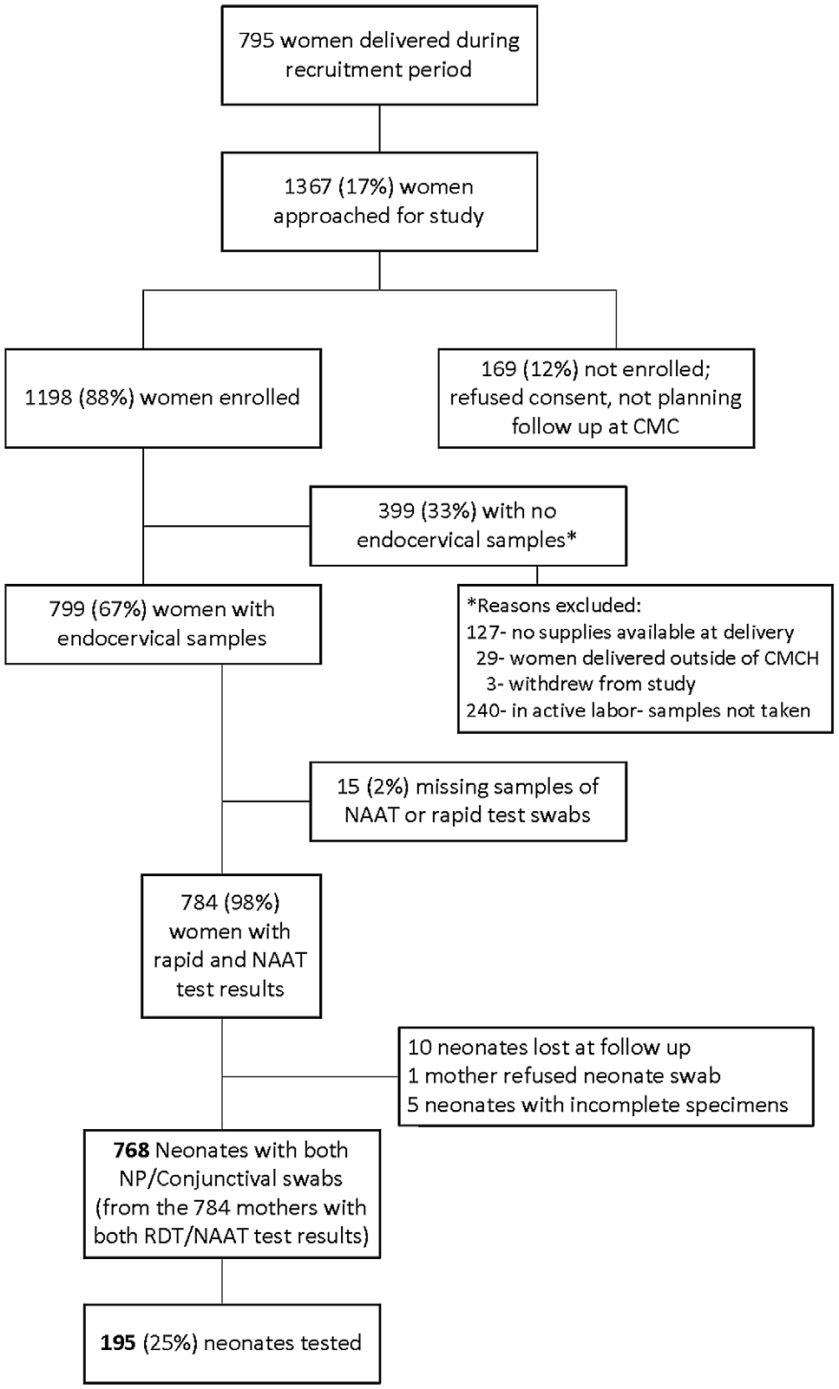
Study Flow Chart. April 2009 to January 2010, 7955 women delivered during the recruitment period. 1198 (88%) women were enrolled; 799 endocervical samples from the 1198 enrolled subjects were collected and data on 784 participants with both RDT and NAAT results are reported.

From April 2009 to January 2010, 7955 women delivered during the recruitment period. Of these 7955 women, a convenience sample of 1367 (17%) pregnant and delivering women was approached for recruitment into the study. Of those, 1198 (88%) women were enrolled; 45% during an antenatal outpatient visit, 55% before a scheduled labor/induction, and <1% in the postpartum ward. Figure 1 shows the study flow diagram.

From the initial 1367 participants approached, 169 delivering women were not enrolled. Of these, 131 women were not enrolled in the study after refusing consent or not planning on follow up care at CMC. Another 38 women did not participate in the study for other reasons. Inquiries were made to determine the reasons for declining; 85% indicated they declined because their family did not want them to participate. Overall, women who declined were similar to the women who did participate in the study in regards to urban/rural residence (p = 0.77), religion (p = 0.47), and gravida (p = 0.1).

Table 2 outlines the demographic characteristics of the enrolled study participants. The mean age of mothers was 25.6 years and 22% (95% CI: 19.7–24.4%) were primigravida. Mean income was 5064 Rupees/month and 50% of mothers had at least a secondary education or higher. The predominant religion of enrolled women was Hinduism which is a reflection of the population composition in Vellore, India. Urban and village residents were of equal distribution. All women enrolled were married; one reported >one sexual partner; and six reported prior STI.

**Table 2:** Baseline characteristics of Enrolled Mothers, Enrolled Tested mothers, and Enrolled and not Tested.

	Enrolled Mothers		Enrolled Tested		Enrolled Not Tested		P-Value of Tested mothers vs. Untested mothers
	N	Percent	N	Percent	N	Percent	
**Age Group**	1198		783		415		**0.03**
18–23 years	384	32.05	231	29.50	153	36.87	
24–29 years	625	52.17	425	54.28	200	48.19	
≥30 years	189	15.78	127	16.22	62	14.94	
**Parity**	1198		783		415		**<0.0001**
Other	935	78.05	649	82.89	286	68.91	
Primigravida	263	21.90	134	17.11	129	31.08	
**Mother’s Education**	1195		780		415		**0.08**
Illiterate	31	2.59	20	2.56	11	2.65	
Primary Education	443	37.07	277	35.51	166	40	
Secondary/Tertiary	293	24.52	183	23.46	110	26.51	
University Diploma	428	35.82	300	38.46	128	30.84	
**Income Group** *	1121		731		390		**0.03**
(<5000 Rs/mo)	795	70.92	499	68.26	296	75.9	
(5000–10,000 Rs/mo)	229	20.43	162	22.16	67	17.18	
(>10,000 Rs/mo)	97	8.65	70	9.58	27	6.92	
**Residence**	1196		781		415		**0.86**
Village	604	50.50	393	50.32	211	50.84	
Urban	592	49.50	388	49.68	204	49.16	
**Religion**	1193		779		414		**0.07**
Hindu	964	80.80	635	81.51	329	79.47	
Muslim	165	13.83	96	12.32	69	16.67	
Christian	62	5.20	47	6.03	15	3.62	
Jain	2	0.17	1	0.13	1	.24	
**History of STI**	1183		775		408		**0.64**
No	1147	96.96	751	96.90	396	97.06	
Yes	6	0.51	3	0.39	3	.74	
Don’t Know	30	2.54	21	2.71	9	2.21	
**Sexual Partners**	1190		775		415		**1**
Only Husband	1189	99.92	774	99.87	415	100	
Others	1	0.08	1	0.13			

* (50Rs = 1 USD).

This table shows that tested mothers were significantly older, multiparous, and higher socio-economic group compared to untested mothers (p = 0.03, p = <0.0001, and p = 0.03; respectively).

We collected 799 endocervical samples from the 1198 enrolled subjects and data on 784 participants with both RDT and NAAT results are reported. 399 enrolled participants (33%) did not have endocervical samples collected after enrollment; see figure 1 for reasons. Tested mothers were significantly older, multiparous, and in the higher socio-economic group compared to untested mothers (p = 0.03, p = <0.0001, and p = 0.03; respectively). Please see table 2. All other demographic were similar. Fifteen (2%) endocervical samples were lost in processing after collection.

### NAAT Versus Rapid Test

The prevalence detected using the NAAT (considered the gold standard for this study) was 0.1% (95% CI: 0–0.38%). The mother with the positive NAAT specimen had no significant characteristics or risk factors. All 71 RDT samples that were positive were considered to be false positives. Thus, compared to NAAT this RDT had a sensitivity of 0%, a specificity of 90%. As anticipated, the statistical analysis by Kappa and Kendall Tau B revealed no agreement between the RDT and NAAT (Table 3). The samples retested independently in an established laboratory confirmed the data found in India, including confirming the single positive. Both sets of laboratories confirmed that there is a low incidence of CT in this population of India.

**Table 3:** Comparison of NAAT test with rapid diagnostic test.

	RDT		
NAAT	Positive	Negative	Total
Positive	0	1	1
Negative	71	712	783
Total	71	713	784
Cohen’s Kappa = −0.0025			
Kendall’s Tau B = −0.0113			

Cohen’s Kappa, which tests the agreement between two tests is negative, showing no agreement. Kendall’s Tau B is also negative, showing very slight negative association (inversion) between the two tests. The association here is very limited.

The prevalence detected using the NAAT (considered the gold standard for this study) was 0.1% (95% CI: 0–0.38%).

### Neonatal Data

There were 811 neonates enrolled which included 12 twin infants. There were 768 newborn specimens (NP and conjunctival) obtained from the neonates of the 784 enrolled mothers who had both NAAT and RDT results reported. During neonatal swab collection, there were 10 neonates who were lost to follow-up due to early discharge, 1 mother refused swab collection from her infant after delivery, and 5 neonates had incomplete swab collections. The neonatal characteristics included 52% born by normal vaginal delivery; mean gestation was 39 weeks, and mean birth weight was 2.99 kilograms (not including twins). Please see Table 4. Only 11% of neonates had newborn problems which included neonatal intensive care admission for risk of sepsis; prematurity, respiratory distress syndrome, gestational diabetes. We expected to have minimal transmission to the neonate since only one mother tested positive by NAAT. Because there was such a low prevalence of mothers with NAAT positive results; we decided to test only 25% of all neonatal samples collected which accounted for 195 samples. All neonatal NAAT specimens, including NP and conjunctival swabs, were negative; including the infant of the mother with the positive endocervical NAAT result. The neonate born to the NAAT positive mother did not have any neonatal problems.

**Table 4:** Neonate Characteristics.

	N	Percent	
**Delivery Type, n = 768**			
Forceps	164	21.35	
C Section	201	26.17	
Normal Vaginal	403	52.47	
**Newborn Problems, n = 768**			
No	681	88.67	
Yes (RDS, Sepsis, IUGR, NICU)	87	11.33	
**Neonate**	**N**	**Mean**	**Std Dev**
**Gestational Age (weeks)**	768	39.0	1.359
**Birth Weight (kilograms)**	762	2.99	0.455

There were 768 newborn specimens (NP and conjunctival) obtained from the neonates of the 784 enrolled mothers who had both NAAT and RDT results reported. This table describes the neonatal characteristics.

## Discussion

This is the largest report from India on *Chlamydia trachomatis* infection in healthy pregnant women in a non-urban setting using NAAT technique. Previous studies have shown the reported chlamydial infection rates in India have ranged as high as 33% to as low as 3.3% [5]–[14] (Table 1). Most of these studies however have focused on high risk population groups in urban settings, potentially could have underestimated prevalence compared to more sensitive NAAT testing [5], [8], [10], [11], [14]. Previous studies in normal pregnant women showed a lower range of infection (3.3–18.8%) using EIA. We used NAAT for confirmatory testing and showed the prevalence of genital chlamydia infections amongst pregnant women to be 0.1% (95% CI: 0–0.38%). Based on this low prevalence it appears that routine prenatal screening in southern India does not appear to be indicated.

In our study, we found that 10% of specimens were positive when evaluated by RDT. We chose the Clearview Chlamydia Test Kit® because it is one of the few FDA approved RDT for CT, and it had reasonable reported sensitivity (53–73%) and specificity (68–99%) on endocervical specimens when compared to culture or DNA probe assay/NAAT [19], [20], [22], [23]. However, in our low prevalence setting the sensitivity and specificity were lower (0% sensitive and 90% specific) and there was a major discrepancy between the RDT and NAAT results. Other studies have also noted lower sensitivity and specificity of RDT and EIA when compared to PCR, especially in low prevalence settings [25], [26].

If the true specificity of the RDT is between 68 and 99% [19]–[23], we would expect a range of 7 to 356 false positives in a sample size of 784 subjects from a population with a prevalence of 3.3%. We observed 71 such results. Similarly, if the true sensitivity is between 58–78%, it is not surprising that the one true positive case would be missed. The package insert states the antibody used in the RDT detects all 15 Chlamydia serovars in addition to *Chlamydia psittaci* [27]. However, cross-reactivity is unlikely as the primary explanation for these results.

In newborns, the CT prevalence was found to be zero. Therefore, the transmission rate from Chlamydia positive mothers to infants could not be characterized because we did not have sufficient power to address this question. Further studies need to be done on pregnant mothers with a higher prevalence of CT infection to assess maternal to neonate transmission in this setting.

There are some limitations to our study. First, due to limited staff, we were only able enroll 17% of delivering women during our study period. However, 88% of women approached were enrolled. Second, the project sample population may not represent the local delivering female population. CMCH is a tertiary, private hospital and some patients may go to a nearby government hospital, or deliver at home. In addition, not all endocervical samples were obtained from recruited participants due to logistical reasons. Few endocervical specimens (n = 2) were also obtained postpartum which may have changed the RDT sensitivity and/or specificity. Tested mothers were significantly older, multiparous, and in the higher socio-economic group versus untested mothers (p = 0.03, p = <0.0001, p = 0.03; respectively). One reason for this finding may be because younger women in the lower socio-economic group delivered their neonate at home (especially if primigravida) or at a nearby government hospital.

In summary, we found that CT infection appears to be relatively rare in women delivering in this private tertiary care hospital in Vellore, India. We found a prevalence of 0.1% (95% CI: 0–0.38%) by NAAT which was much lower than that noted in other international studies including pregnant women [28]–[31]. However, Joyee *et al.* [8] found a prevalence of 1.1% (95% CI: 0.4–1.8%) in the healthy female population of Tamil Nadu using urine NAAT. Their result of a low CT prevalence supports our findings of a very low rate of CT infection in our similar population. Furthermore, NAAT proved to be superior in the diagnosis of CT than the RDT in a low prevalence setting.

The local practice is to not perform prenatal screening in healthy pregnant women in this population based on evidence from twenty years ago which used older diagnostic methods. We conclude that our current study justifies that routine prenatal screening in this population would not be recommended given the low prevalence. However, future studies are important to reassess prevalence as sexual practices may change within this culture. Outpatient pre-natal testing may provide better information in all SES strata.

## References

[1] World Health Organization (2010). Sexually Transmitted Diseases - *Chlamydia trachomatis,* 2010.. http://www.who.int/vaccine_research/diseases/soa_std/en/index1.html.

[2] Chen CJ, Wu KG, Tang RB, Yuan HC, Soong WJ (2007). Characteristics of *Chlamydia trachomatis* infection in hospitalized infants with lower respiratory tract infection.. J Microbiol Immunol Infect..

[3] Hammerschlag MR (1989). Chlamydial infections.. J Pediatr.

[4] Rosenman MB, Mahon BE, Downs SM, Kleiman MB (2003). Oral erythromycin prophylaxis vs watchful waiting in caring for newborns exposed to *Chlamydia trachomatis*.. Arch Pediatr Adolesc Med.

[5] Alexander R, Mathai E, Nayyar V, Mathew M, Jasper P (1993). Low prevalence of chlamydial endocervical infection in antenatal south Indian women.. Genitourin Med.

[6] Becker M, Stephen J, Moses S, Washington R, Maclean I (2010). Etiology and determinants of sexually transmitted infections in Karnataka state, south India.. Sex Transm Dis.

[7] Brabin L, Gogate A, Gogate S, Karande A, Khanna R (1998). Reproductive tract infections, gynaecological morbidity and HIV seroprevalence among women in Mumbai, India.. Bull World Health Organ.

[8] Joyee AG, Thyagarajan SP, Rajendran P, Hari R, Balakrishnan P (2004). *Chlamydia trachomatis* genital infection in apparently healthy adult population of Tamil Nadu, India: a population-based study.. Int J STD AIDS.

[9] Joyee AG, Thyagarajan SP, Reddy EV, Venkatesan C, Ganapathy M (2005). Genital chlamydial infection in STD patients: its relation to HIV infection.. Indian J Med Microbiol..

[10] Malik A, Jain S, Rizvi M, Shukla I, Hakim S (2009). *Chlamydia trachomatis* infection in women with secondary infertility.. Fertil Steril..

[11] Mania-Pramanik J, Meherji PK, Gokral JS, Donde UM (2001). *Chlamydia trachomatis* infection in an urban setting.. Sex Transm Infect.

[12] Rastogi S, Das B, Salhan S, Mittal A (2003). Effect of treatment for *Chlamydia trachomatis* during pregnancy.. Int J Gynaecol Obstet..

[13] Singh V, Salhan S, Das BC, Mittal A (2003). Predominance of *Chlamydia trachomatis* serovars associated with urogenital infections in females in New Delhi, India.. J Clin Microbiol..

[14] Singh V, Sehgal A, Satyanarayana L, Gupta MM, Parashari A (1995). Clinical presentation of gynecologic infections among Indian women.. Obstet Gynecol.

[15] Paul VK, Singh M, Gupta U, Buckshee K, Bhargava VL (1999). *Chlamydia trachomatis* infection among pregnant women: prevalence and prenatal importance.. Natl Med J India.

[16] Berg A (2001). Screening for chlamydial infection: recommendations and rationale.. Am J Prev Med.

[17] U.S. Preventive Services Task Force (2007). *Screening for Chlamydial Infection: U.S. Preventive Services Task Force Recommendation Statement*. First published in Ann Intern Med 2007;147: 128–33.. http://www.uspreventiveservicestaskforce.org/uspstf07/chlamydia/chlamydiars.htm.

[18] Centers for Disease Control and Prevention (2008). Sexually Transmitted Disease Surveillance, Atlanta, GA: U.S. Department of Health and Human Services; November 2009.

[19] Blanding J, Hirsch L, Stranton N, Wright T, Aarnaes S (1993). Comparison of the Clearview Chlamydia, the PACE 2 assay, and culture for detection of *Chlamydia trachomatis* from cervical specimens in a low-prevalence population.. J Clin Microbiol.

[20] Hislop J, Quayyum Z, Flett G, Boachie C, Fraser C (2010). Systematic review of the clinical effectiveness and cost-effectiveness of rapid point-of-care tests for the detection of genital chlamydia infection in women and men.. Health Technol Assess.

[21] Kluytmans JA, Goessens WH, Mouton JW, van Rijsoort-Vos JH, Niesters HG (1993). Evaluation of Clearview and Magic Lite tests, polymerase chain reaction, and cell culture for detection of *Chlamydia trachomatis* in urogenital specimens.. J Clin Microbiol.

[22] Saison F, Mahilum-Tapay L, Michel CE, Buttress ND, Nadala EC (2007). Prevalence of *Chlamydia trachomatis* infection among low- and high-risk Filipino women and performance of Chlamydia rapid tests in resource-limited settings.. J Clin Microbiol.

[23] Yin YP, Peeling RW, Chen XS, Gong KL, Zhou H (2006). Clinic-based evaluation of Clearview Chlamydia MF for detection of *Chlamydia trachomatis* in vaginal and cervical specimens from women at high risk in China.. Sex Transm Infect.

[24] Livengood CH, Wrenn JW (2001). Evaluation of COBAS AMPLICOR (Roche): accuracy in detection of *Chlamydia trachomatis* and *Neisseria gonorrhoeae* by coamplification of endocervical specimens.. J Clin Microbiol.

[25] Horner P, Skidmore S, Herring A, Sell J, Paul I (2005). Enhanced enzyme immunoassay with negative-gray-zone testing compared to a single Nucleic Acid Amplification Technique for community-based chlamydial screening of men.. J Clin Microbiol.

[26] Lauderdale TL, Landers L, Thorneycroft I, Chapin K (1999). Comparison of the PACE 2 assay, two amplification assays, and Clearview EIA for detection of *Chlamydia trachomatis* in female endocervical and urine specimens.. J Clin Microbiol.

[27] The Inverness Medical Group of Companies (2006). http://www.clearview.com/pdf/CV_Ch211BBB_EN.pdf.

[28] El-Qouqa IA, Shubair ME, Al Jarousha AM, Sharif FA (2009). Prevalence of *Chlamydia trachomatis* among women attending gynecology and infertility clinics in Gaza, Palestine.. Int J Infect Dis.

[29] Sturm AW, Wilkinson D, Ndovela N, Bowen S, Connolly C (1998). Pregnant women as a reservoir of undetected sexually transmitted diseases in rural South Africa: implications for disease control.. Am J Public Health.

[30] Wessel HF, Herrmann B, Dupret A, Moniz F, Brito C (1998). Genital infections among antenatal care attendees in Cape Verde.. Afr J Reprod Health.

[31] Yongjun T, Samuelson J, Qingsheng D, Ali MM, Li X (2009). The prevalence of sexually transmitted and other lower reproductive tract infections among rural women in Sichuan Province, China.. Southeast Asian J Trop Med Public Health.

